# Ginkgolide B protects retinal pigment epithelial cells from H_2_O_2_-induced oxidative stress and apoptosis by activating the AMPK/Nrf2 pathway

**DOI:** 10.3389/fmed.2026.1755596

**Published:** 2026-07-24

**Authors:** Bei Zhan, Ran Xia, Liming Tao

**Affiliations:** 1 Department of Ophthalmology, The Second Hospital Affiliated to Anhui Medical University, Hefei, China; 2 Department of Ophthalmology, Anhui No.2 Provincial People’s Hospital, Hefei, China

**Keywords:** AMPK/Nrf2, apoptosis, Ginkgolide B, oxidative stress, retinal pigment epithelial cells

## Abstract

**Objective:**

To investigate whether Ginkgolide B (GB) protects retinal pigment epithelial cells (RPEs) from hydrogen peroxide (H_2_O_2_)-induced oxidative stress and apoptosis, and to explore the potential involvement of the adenosine monophosphate-activated protein kinase *α* (AMPKα)/nuclear factor erythroid 2-related factor 2 (Nrf2) pathway.

**Methods:**

Human RPEs were divided into the following groups: control, model (200 μmol/L H₂O₂ for 24 h), GB + H₂O₂, GB + H₂O₂ + Compound C (an AMPK inhibitor), H₂O₂ + Compound C, and GB alone. Cell viability, apoptosis, antioxidant parameters, and AMPKα/Nrf2 expression were assessed using the Cell Counting Kit-8 (CCK-8) assay, flow cytometry, colorimetric biochemical assays, transmission electron microscopy (TEM), quantitative polymerase chain reaction (PCR), and Western blot.

**Results:**

TEM showed that GB attenuated H₂O₂-induced mitochondrial swelling and cristae disruption, which were reversed by Compound C. Compared with the model group, the GB + H₂O₂ group exhibited increased cell viability, decreased apoptosis, elevated activities of catalase (CAT), glutathione peroxidase (GPx), and superoxide dismutase (SOD), as well as increased glutathione (GSH) content, and reduced malondialdehyde (MDA) (all *p* < 0.05), though protein carbonyl (PC) levels showed no significant amelioration. All protective effects were attenuated by Compound C. In the GB-alone group, the percentage of apoptotic cells, Nrf2 mRNA expression, and all other parameters did not differ significantly from those in the control group.

**Conclusion:**

GB was associated with protection of RPEs against H₂O₂-induced oxidative stress and apoptosis. Because these protective effects were attenuated by pharmacological inhibition of AMPK, the findings suggest that AMPK/Nrf2 signaling may contribute to the cytoprotective actions of GB. However, additional mechanistic studies are required to confirm the involvement of this pathway.

## 1 Introduction

Age-related macular degeneration (AMD) is a leading cause of blindness among the elderly worldwide. Recent epidemiological analysis in the United States indicates that the crude prevalence of early AMD in people aged 40 and older reached 11.64% in 2020, with late AMD at approximately 0.94%, showing significant variations by age, sex, and race (1). A study also suggests that the number of AMD patients will increase by about 50% around 2020 due to population aging, with over 7 million people at potential risk for early lesions (2). These data highlight the urgent need for effective interventions to address the public health burden of AMD and associated damage to retinal pigment epithelial cells (RPEs).

Substantial clinical and experimental evidence indicates that prolonged exposure of RPEs to high levels of reactive oxygen species (ROS) disrupts the antioxidant defense system, leading to cellular dysfunction and apoptosis, thereby driving the onset and progression of AMD (3). Specifically, nuclear factor erythroid 2-related factor 2 (Nrf2) plays a central role in regulating antioxidant enzyme expression in RPEs. Nrf2-deficient mice develop AMD-like pathologies with aging, including RPE degeneration, lipofuscin accumulation, and choroidal neovascularization, further confirming the indispensable role of Nrf2 in antioxidant defense in RPEs (4).

Intervention studies targeting oxidative damage in RPEs have made some progress. Genipin can enhance RPEs’ tolerance to H₂O₂ by upregulating Nrf2 and its downstream antioxidant enzymes, such as heme oxygenase-1 (HO-1) and NAD(P)H quinone dehydrogenase 1 (NQO1). However, this effect is limited to the Nrf2 pathway and does not involve regulation of energy metabolism (5). Berberine significantly inhibited H₂O₂-induced RPE apoptosis by activating the phosphorylation of AMP-activated protein kinase *α* (AMPKα), suggesting the protective potential of AMPKα in RPE antioxidant systems; however, it did not simultaneously evaluate the Nrf2 pathway (6). Metformin, an AMPKα activator, promoted autophagy and enhanced the antioxidant capacity of RPEs, but its direct activation of Nrf2 has not been confirmed, and its comprehensive protective effects *in vitro* require further validation (7). Furthermore, Ginkgolide B (GB) has demonstrated antioxidant activity in vascular endothelial cells by inhibiting NADPH oxidase 4 (NOX4) expression and reducing ROS production, but direct experimental data in retinal cells are lacking, limiting its prospects in ophthalmology (8). While these studies alleviated oxidative stress in RPEs to varying degrees, they generally failed to regulate multiple signaling pathways and did not achieve synergistic upregulation of both AMPKα and Nrf2.

Given these limitations, this study aims to systematically evaluate the dual regulatory effect of GB in RPEs. Existing research shows that GB can activate AMPKα and inhibit oxidative stress and apoptosis via the AMPKα-Nrf2 axis in astrocytes (9). Its antioxidant effects in vascular endothelial cells further suggest its potential to regulate ROS (8). Therefore, we sought to investigate whether GB treatment is associated with changes in AMPKα and Nrf2 signaling in an H₂O₂-induced RPE oxidative injury model, thereby providing an innovative strategy for AMD intervention that combines regulation of energy metabolism and antioxidant defense.

## 2 Materials and methods

### 2.1 Experimental materials and instruments

Human retinal pigment epithelial cells (ARPE-19 cell line) used in this study were purchased from Shanghai Hongshun Biotechnology Co., Ltd.^1^ These cells are derived from the ARPE-19 cell line. Cells were cultured in Dulbecco’s modified Eagle medium/nutrient mixture F-12 (DMEM/F12) (Servicebio, G4515-500ML) supplemented with 10% fetal bovine serum (FBS) and 1% penicillin/streptomycin, and routinely maintained in a 37 °C, 5% CO₂ incubator. Main experimental reagents included cell counting kit-8 (CCK-8) (Servicebio, G4103-5ML), phosphate-buffered saline (PBS) (Servicebio, G0002), RNA easy fast animal tissue/cell total RNA extraction kit (TIANGEN, DP451-TA), catalase (CAT) colorimetric assay kit (Elabscience, E-BC-K031-M), glutathione peroxidase (GPx) assay kit (Beyotime, S0056), total superoxide dismutase (SOD) activity assay kit (WST-8 method) (Beyotime, S0101S), reduced glutathione (GSH) content assay kit (Solarbio, BC1175), malondialdehyde (MDA) content assay kit (Solarbio, BC0020), protein carbonyl (PC) content assay kit (Solarbio, BC1270), Beyort™ II first-strand cDNA synthesis kit (Beyotime, D7168M), and beyofast™ SYBR Green qPCR mix (Beyotime, D7262-25 mL). Key instruments included a mini centrifuge (Servicebio, D1008E), a decolorizing shaker (Servicebio, TSY-B), pipettes (Dragon, KE0003087/KA0056573), an upright fluorescence microscope (Nikon Eclipse C1, Japan), a gel imaging system (Tanon 5,200, Shanghai, China), and a real-time quantitative PCR instrument (Bio-Rad, Chromo4).

### 2.2 Experimental methods

#### 2.2.1 Cell culture and experimental grouping

RPEs were rapidly thawed in a 37 °C water bath with gentle agitation until completely thawed. The cryopreservation medium was aspirated into a 15 mL centrifuge tube under a sterile bench, mixed with 1 mL complete medium, and centrifuged at 1000 rpm for 5 min at room temperature. The supernatant was discarded, and the cell pellet was resuspended in 1 mL complete medium. Cells were then seeded into culture flasks (T-25) and incubated at 37 °C with 5% CO₂. The culture medium was replaced every 2–3 days. When cell confluence reached 80–90%, subculture was performed: old medium was discarded, cells were washed twice with 1 mL PBS, PBS was discarded, and 1 mL of 0.25% trypsin containing ethylenediaminetetraacetic acid (EDTA) was added to digest for approximately 5 min. Upon observing cell rounding under the microscope, 1 mL medium was added to terminate digestion. Cells were gently pipetted to detach, centrifuged at 1000 rpm for 5 min at room temperature, the supernatant was discarded, and the pellet was resuspended in 2 mL complete medium and allocated to new 100-mm culture dishes, followed by the addition of 8 mL of complete medium to each dish (final volume of 10 mL). For plating, cells were seeded at densities of 8 × 10^4^ cells/well in 6-well plates, 4 × 10^4^ cells/well in 12-well plates, 2 × 10^4^ cells/well in 24-well plates, and 5 × 10^3^ cells/well in 96-well plates, and experiments were conducted after 18–24 h of culture.

A unified timeline was applied to ensure identical treatment timing: the start of treatment was defined as 0 h. At 0 h, Compound C (water-soluble) or an equal volume of complete medium was added; at 1 h, GB (in DMSO) or an equal volume of pure DMSO was added; at 2 h, H₂O₂ (in complete medium) or complete medium alone was added, followed by 24 h of incubation. The experimental groups were as follows: (1) Control Group (CG): cells received an equal volume of complete medium at 0 h, an equal volume of DMSO at 1 h, and an equal volume of complete medium without H₂O₂ at 2 h, then were cultured for 24 h, serving as the normal control. (2) H₂O₂ Model Group (MG): cells received complete medium at 0 h, DMSO at 1 h, and 200 μM H₂O₂ in complete medium at 2 h, followed by a 24 h incubation to induce oxidative stress, serving as the disease (model) control. (3) Ginkgolide B + H₂O₂ Group (GHG): cells received complete medium at 0 h, were pretreated with GB (10 μM) at 1 h, and then exposed to 200 μM H₂O₂ in complete medium at 2 h for 24 h, designed to evaluate the protective effect of GB against H₂O₂-induced oxidative damage. (4) Ginkgolide B + H₂O₂ + Compound C Group (GHG + ComC): cells were pretreated with Compound C (an AMPK inhibitor, 10 μM) at 0 h; without washing, GB (10 μM) was added directly at 1 h, and cells were subsequently exposed to 200 μM H₂O₂ in complete medium at 2 h for 24 h, ensuring the continuous presence of both Compound C and GB during H₂O₂ exposure. This group was used to verify whether the AMPK pathway is necessary for GB-mediated cytoprotection; if the protective effects were abolished by Compound C, it would confirm that GB acts through the AMPK pathway. (5) H₂O₂ + Compound C Group (MG + ComC): cells were pretreated with Compound C (10 μM) at 0 h, received an equal volume of DMSO at 1 h, and were then exposed to 200 μM H₂O₂ in complete medium at 2 h for 24 h, evaluating the effect of AMPK inhibition alone under oxidative stress conditions. (6) Ginkgolide B-only Group (GOG): cells received complete medium at 0 h, were treated with GB (10 μM) at 1 h, and were then given an equal volume of complete medium without H₂O₂ at 2 h, incubated for 24 h, and used to assess the effect of GB on normal cells and to rule out its potential cytotoxicity.

The concentration of 200 μM H₂O₂ has been confirmed in multiple studies (5, 10) to be optimal for inducing oxidative damage in RPEs and was therefore selected to establish the model. GB was dissolved in dimethyl sulfoxide (DMSO) to prepare a 10 mM stock solution, which was stored at −20 °C. For experiments, the stock solution was diluted in complete culture medium to achieve a final concentration of 10 μM. In 100-mm culture dishes containing 10 mL of medium, 10 μL of GB stock solution was added. The final concentration of DMSO was 0.1%, which has no detectable effect on ARPE-19 cell viability. Control cells received an equivalent volume of DMSO.

#### 2.2.2 Cytotoxicity assay and oxidative stress model establishment

Cells were seeded at 5 × 10^3^ cells/well in a 96-well plate. After 24 h of attachment culture, the original medium was discarded, and culture medium containing 200 μM H₂O₂ was added to each well. Groups included a blank control and 6-, 12-, and 24-h treatment groups. Cell viability was determined using the CCK-8 assay: 10 μL of CCK-8 solution was added to each well, the plate was incubated at 37 °C for 2 h, and the optical density (OD) at 450 nm was measured using a microplate reader. Based on the cytotoxicity results, treatment with 200 μM H₂O₂ for 24 h, which reduced cell viability to approximately 50%, was selected as the condition for establishing the oxidative stress model for subsequent experiments.

#### 2.2.3 Flow cytometry for apoptosis detection

Apoptosis was assessed using an Annexin V-FITC/PI Apoptosis Detection Kit (Beijing Solarbio Science & Technology Co., Ltd.; Catalogue no. CA1020) according to the manufacturer’s instructions. After 24 h of treatment, both floating and adherent cells were collected. Briefly, culture supernatants were first transferred to centrifuge tubes to retain detached cells. Adherent cells were gently washed twice with cold PBS and digested with 0.25% trypsin without EDTA. The digestion was terminated with complete medium, and cells were combined with the corresponding supernatants and centrifuged at 1500 rpm for 5 min at 4 °C. The cell pellet was washed twice with cold PBS and resuspended in 500 μL of 1 × binding buffer at a concentration of approximately 1 × 10^6^ cells/mL.

Subsequently, 5 μL Annexin V-FITC and 5 μL propidium iodide (PI, 50 μg/mL) were added to each sample, resulting in final concentrations of approximately 0.5 μg/mL Annexin V-FITC and 0.5 μg/mL PI. Cells were incubated for 10 min at room temperature in the dark and immediately analyzed by flow cytometry using a BD FACSCalibur (BD Biosciences, San Jose, CA, USA), and the data were analyzed with FlowJo (version 10.7). Fluorescence compensation was performed using single-stained controls before data acquisition.

#### 2.2.4 Detection of oxidative stress markers

Intracellular antioxidant enzyme activities and oxidative stress markers were measured using commercial assay kits according to the manufacturers’ instructions. After the respective treatments, cells were harvested and lysed in ice-cold lysis buffer. The lysates were centrifuged at 4 °C and 12,000 rpm for 10 min to remove debris, and the supernatants were collected for analysis. Total protein concentration was determined using a bicinchoninic acid (BCA) Protein Assay Kit (Beyotime, Shanghai, China) and was used to normalize readouts. For enzyme activity assays, including catalase (CAT), glutathione peroxidase (GPx), and total superoxide dismutase (SOD), samples were standardized by loading a fixed amount of total protein (20–50 μg) per reaction. The fixed sample loading volumes required by each kit (manufacturer’s microplate protocol) were: 40 μL for the CAT assay (Elabscience, Cat. No. E-BC-K031-M), 10 μL for the GPx assay (Beyotime, Cat. No. S0056), and 20 μL for the total SOD assay (Beyotime, Cat. No. S0101S; water-soluble tetrazolium salt-8, WST-8 method). To achieve the target protein input within these fixed loading volumes, the required volume of each lysate was calculated based on its BCA-determined concentration. If the required volume was less than or equal to the fixed loading volume, the lysate was used without pre-dilution (pre-dilution factor = 1), and the remaining volume was made up with the corresponding assay buffer to reach the fixed loading volume. If the required volume exceeded the fixed loading volume, the lysate was pre-diluted with assay buffer (2-fold; pre-dilution factor = 2), and the volume was recalculated accordingly. For reduced glutathione (GSH; Solarbio, Cat. No. BC1175; 100 μL of deproteinized supernatant) and for malondialdehyde (MDA; Solarbio, Cat. No. BC0020; 100 μL of lysate) and protein carbonyl (PC; Solarbio, Cat. No. BC1270; 100 μL of lysate), sample volumes were fixed according to the manufacturers’ protocols, and the results were normalized to the BCA-determined total protein level measured from the corresponding lysate. All colorimetric assays were performed following the manufacturers’ 96-well microplate procedures (typically 200 μL per well). Subsequently, antioxidant parameters, including CAT, GPx, total SOD, and GSH, were assessed as follows. CAT activity was determined using a colorimetric method. In this method, CAT-catalyzed H₂O₂ decomposition was stopped by ammonium molybdate. The remaining H₂O₂ reacted with a chromogen to form a pale yellow complex, measured at 405 nm. GPx activity was measured via a coupled enzymatic reaction. It was quantified by the rate of decrease in the absorbance of reduced nicotinamide adenine dinucleotide phosphate (NADPH) at 340 nm. Total SOD activity was assessed using the WST-8 method. WST-8 reacts with superoxide anions to produce a water-soluble formazan dye. SOD activity was inversely proportional to the formation of this product and was measured at 450 nm. GSH content was quantified based on its reaction with 5,5′-dithiobis(2-nitrobenzoic acid) (DTNB). This produces a yellow product detected at 412 nm. Oxidative damage markers, including MDA and PC, were evaluated. MDA content was measured using the thiobarbituric acid (TBA) colorimetric method. In this method, MDA reacts with TBA at high temperature under acidic conditions to form a red adduct, which is detected at 532 nm. PC content was determined using the 2,4-dinitrophenylhydrazine (DNPH) method. Here, carbonyl groups react with DNPH to form a reddish-brown precipitate, later dissolved and measured at 370 nm.

#### 2.2.5 Transmission electron microscopy observation

After 24 h of the corresponding treatment, the medium was discarded, and the cells were washed twice with PBS. The cells were digested with 0.25% trypsin for 2–3 min. Digestion was terminated with complete medium, and the cells were collected by centrifugation at 1000 rpm for 5 min and washed twice with PBS. The supernatant was discarded, and the cells were fixed with 2.5% glutaraldehyde overnight at 4 °C. The cell pellets were rinsed, post-fixed with 1% osmium tetroxide for 2 h, dehydrated through a graded ethanol series, substituted with acetone, infiltrated and embedded in epoxy resin, sectioned using an ultramicrotome, double-stained with uranyl acetate and lead citrate, and finally observed under a transmission electron microscope to capture ultrastructural images.

#### 2.2.6 Real-time quantitative PCR (qPCR) for gene expression

After 24 h of treatment, total RNA was extracted using the RNeasy Fast Kit, and RNA concentration and purity were measured. According to the instructions of the BeyoRT™ II cDNA First Strand Synthesis Kit, 1 μg of RNA was reverse-transcribed into cDNA. Using cDNA as a template, amplification was performed with BeyoFast™ SYBR Green qPCR Mix. The reaction system (20 μL) consisted of 10 μL TB Green Premix Ex Taq II, 1 μL forward primer (10 μM), 1 μL reverse primer (10 μM), 2 μL cDNA template, and 6 μL sterile water. The qPCR program was as follows: pre-denaturation at 95 °C for 30 s; 40 cycles of 95 °C for 5 s and 57.5 °C for 34 s; followed by a melt curve stage (95 °C for 15 s, 60 °C for 60 s, 95 °C for 15 s). The 2^^−ΔΔCt^ method was used to calculate the relative mRNA expression levels of AMP-activated protein kinase *α* (AMPKα) and Nrf2, with glyceraldehyde-3-phosphate dehydrogenase (GAPDH) used as the internal reference gene. The sequences of the PCR primers are listed in Table 1.

**Table 1:** Primer sequences utilized for real-time quantitative polymerase chain reaction analysis.

Gene name	Primer direction	Primer sequence (5′→3′)
GAPDH	Forward Primer	5′-TGGCCTTCCGTGTTCCTAC -3′
GAPDH	Reverse Primer	5′-GAGTTGCTGTTGAAGTCGCA −3′
AMPKα	Forward Primer	5’-TTGAAACCTGAAAATGTCCTGCTT-3′
AMPKα	Reverse Primer	5’-GGTGAGCCACAACTTGTTCTT-3′
Nrf2	Forward Primer	5′-TTGGCAGAGACATTCCCATTTGTA-3′
Nrf2	Reverse Primer	5′-GAGCTATCGAGTGACTGAGCCTG-3′

#### 2.2.7 Western blot analysis of protein expression

After 24 h of the corresponding treatments, cells were washed twice with ice-cold PBS and lysed on ice in RIPA lysis buffer (Beyotime, Cat. No. P0013B) supplemented with 1 mM PMSF (Beyotime, Cat. No. ST506) and a protease/phosphatase inhibitor cocktail (Beyotime, Cat. No. P1045) for 30 min. Lysates were centrifuged at 13,000 × g for 15 min at 4 °C, and the supernatants were collected as total protein extracts. Protein concentration was determined using a BCA Protein Assay Kit (Beyotime, Cat. No. P0012).

Equal amounts of protein (30 μg per lane) were mixed with 5× SDS–PAGE loading buffer (Beyotime, Cat. No. P0015L), denatured at 100 °C for 10 min, and separated on 12% SDS–PAGE gels. Proteins were transferred onto PVDF membranes (Millipore, Cat. No. IPVH00010) using a wet-transfer system at 300 mA for 90 min at 4 °C. Membranes were blocked in 5% non-fat milk (Beyotime, Cat. No. P0216-300 g) prepared in TBST (20 mM Tris–HCl, pH 7.6, 150 mM NaCl, 0.1% Tween-20) for 1 h at room temperature.

Blotting strategy and antibody incubations (two-step immunoblotting): AMPKα and Nrf2 were analyzed on two separate, parallel immunoblots (i.e., independent gels and PVDF membranes; one membrane for AMPKα and one membrane for Nrf2). On each membrane, the target protein and the loading control (GAPDH) were detected on the same membrane, and no membrane stripping/reprobing was performed. Specifically, the AMPKα membrane was incubated overnight at 4 °C with a primary antibody cocktail containing rabbit anti-AMPKα (Cell Signaling Technology, Cat. No. 2532S; 1:1000) and mouse anti-GAPDH (Proteintech, Cat. No. 60004-1-Ig; 1:5000). In parallel, the Nrf2 membrane was incubated overnight at 4 °C with a primary antibody cocktail containing rabbit anti-Nrf2 (Abcam, Cat. No. ab62352; 1:1000) and mouse anti-GAPDH (Proteintech, Cat. No. 60004-1-Ig; 1:5000).

Following primary antibody incubation, membranes were washed three times with TBST (10 min each) and then incubated for 1 h at room temperature with a secondary antibody cocktail diluted in TBST containing 5% non-fat milk: HRP-conjugated goat anti-rabbit IgG (Cell Signaling Technology, Cat. No. 7074S; 1:5000) and HRP-conjugated goat anti-mouse IgG (Cell Signaling Technology, Cat. No. 7076S; 1:5000). Primary and secondary antibody incubations were performed sequentially (primary incubation → washes → secondary incubation); primary and secondary antibodies were not co-incubated in a single step. After secondary incubation, membranes were washed three times with TBST (10 min each).

Immunoreactive bands were visualized using an ECL substrate (Thermo Fisher Scientific, Cat. No. 32106) and captured with a Tanon 5,200 system. Band intensities were quantified using ImageJ (version 1.53), and target protein signals were normalized to the corresponding GAPDH signal from the same membrane.

### 2.3 Statistical analysis

All experiments were independently performed at least three times (n = 3 per group). Data are presented as the mean ± standard deviation (SD). Prior to statistical analysis, data normality was assessed using the Shapiro–Wilk test, and homogeneity of variance was evaluated using Levene’s test. As the data met the assumptions of normality and homogeneity of variance (*p* > 0.05), comparisons among multiple groups were performed using one-way analysis of variance (ANOVA) followed by Tukey’s *post hoc* test. A *p* value < 0.05 was considered statistically significant. Statistical analyses were conducted using SPSS software (version 26.0).

## 3 Results

### 3.1 Transmission electron microscopy

**Figure 1:**
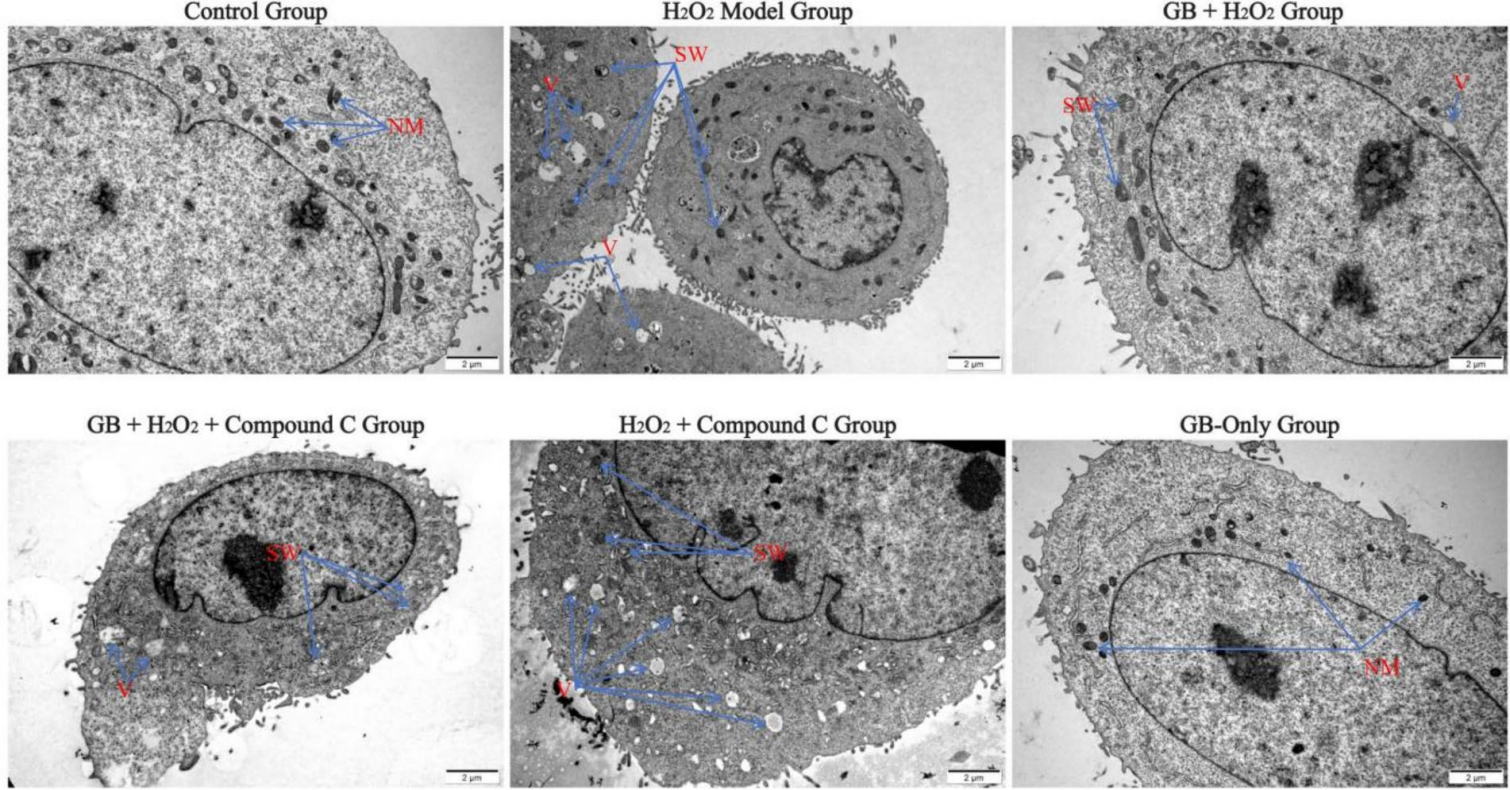
Transmission electron micrograph of retinal pigment epithelium mitochondria (Scale bar: 2 μm). All images within each figure were captured at the same magnification under identical imaging conditions. No artificial enlargement or selective cropping was performed. NM, Normal mitochondria; SW, Swollen mitochondria; V, Vacuolization.

**Figure 2:**
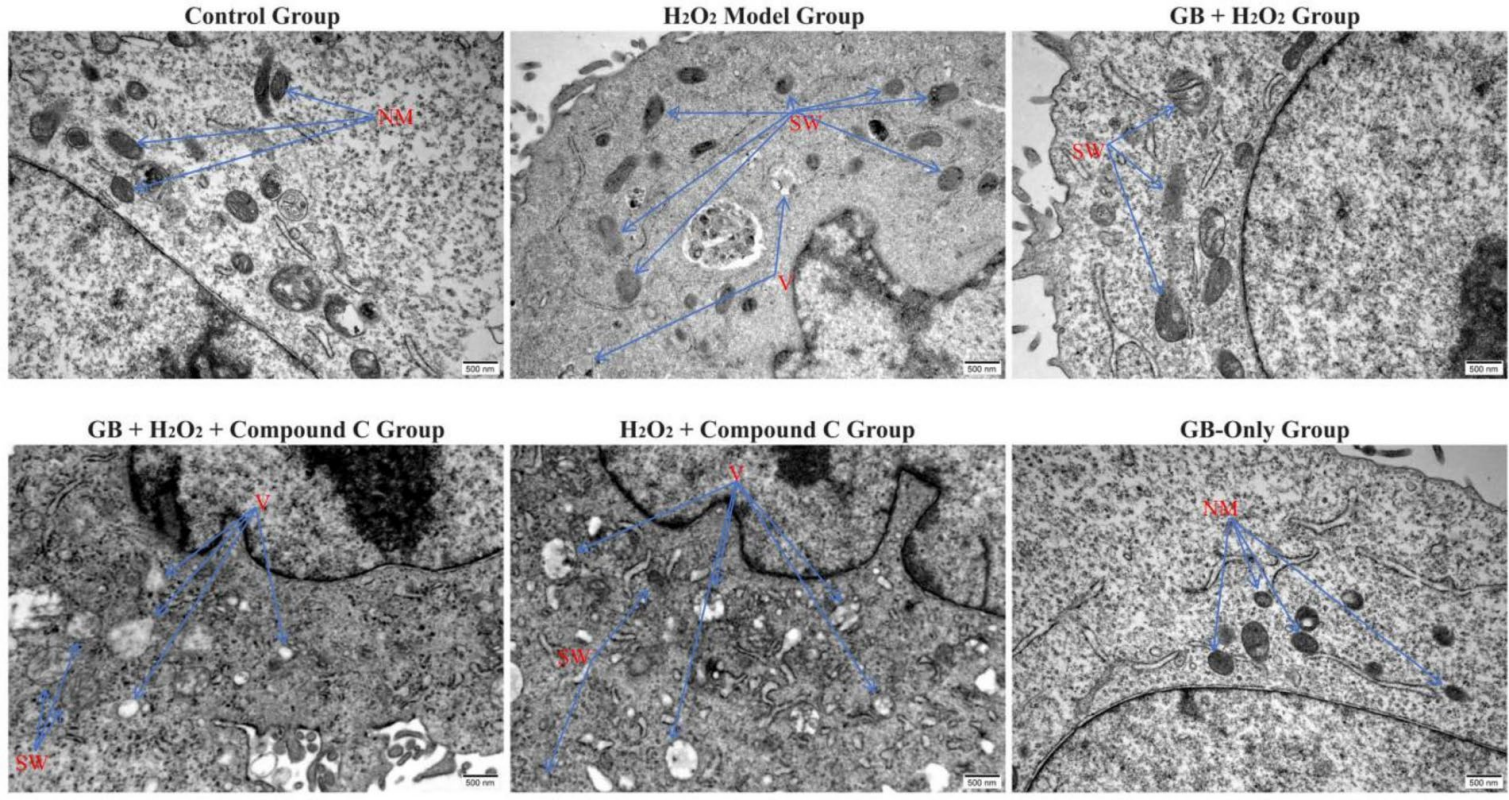
Transmission electron micrograph of retinal pigment epithelium mitochondria (Scale bar: 500 nm). All images within each figure were captured at the same magnification under identical imaging conditions. No artificial enlargement or selective cropping was performed. NM, Normal mitochondria; SW, Swollen mitochondria; V, Vacuolization.

Transmission electron microscopy revealed distinct ultrastructural changes among the experimental groups. In the control group, mitochondria displayed normal morphology, characterized by typical rod- or oval-shaped structures, a uniform matrix, and no obvious swelling or vacuolization. Conversely, in the H₂O₂ model group, intense H₂O₂-induced oxidative stress caused severe mitochondrial damage. This damage was characterized by marked swelling, decreased matrix electron density, a transition toward a spherical shape, and the appearance of vacuolated areas, consistent with mitochondrial injury under oxidative stress. In the GB + H₂O₂ group, GB pretreatment substantially attenuated these injuries. Most mitochondria maintained relatively normal morphology; only a few exhibited mild swelling, and the number of damaged mitochondria was markedly lower than in the H₂O₂ model group. However, in the GB + H₂O₂ + Compound C group, the addition of the AMPKα inhibitor Compound C reversed GB’s protective effects. Mitochondria in this group once again showed pronounced swelling and spherical transformation, and the extent of ultrastructural damage was comparable to that in the H₂O₂ model group. Morphologically, this indicates that blockade of the AMPKα pathway largely abolished GB’s mitochondrial protective effect. In the H₂O₂ + Compound C group, H₂O₂ injury combined with Compound C further aggravated mitochondrial damage compared with the H₂O₂ model group, manifesting as more extensive vacuolization and occasional signs of membrane disruption. Finally, in the GB-alone group, mitochondria showed no apparent abnormalities compared with the control group. The ultrastructure remained largely intact, further confirming that GB is non-toxic to cells under normal conditions, as shown in Figures 1, 2.

### 3.2 Levels of antioxidant enzymes and oxidative stress parameters in RPEs

One-way ANOVA revealed significant differences among the six groups for all antioxidant and oxidative stress parameters (CAT, GPx, SOD, GSH, MDA, and PC; all *p* < 0.05; Table 2).

**Table 2:** Levels of antioxidant enzymes and oxidative stress parameters in RPEs.

Groups	CAT (U/mg prot)	GPx (mU/mg prot)	SOD (U/mg prot)	GSH (μg/mg prot)	MDA (nmol/mg prot)	PC (μmol/mg prot)
CG	10.92 ± 0.80 ^c^	120.67 ± 3.92 ^c^	87.65 ± 2.94 ^c^	22.09 ± 2.39 ^c^	7.51 ± 0.45 ^a^	0.0118 ± 0.0005 ^a^
MG	4.47 ± 0.65 ^a^	38.25 ± 6.55 ^a^	37.61 ± 8.00 ^a^	6.79 ± 0.87 ^a^	21.96 ± 0.49 ^c^	0.0165 ± 0.0047 ^ab^
GHG	8.33 ± 0.66 ^b^	83.31 ± 10.52 ^b^	72.05 ± 2.13 ^b^	14.65 ± 1.20 ^b^	12.65 ± 2.19 ^b^	0.0160 ± 0.0011 ^ab^
GHG + Com C	4.80 ± 0.73 ^a^	42.00 ± 7.35 ^a^	40.00 ± 6.12 ^a^	7.50 ± 1.22 ^a^	20.50 ± 2.45 ^c^	0.0170 ± 0.0012 ^ab^
MG + Com C	3.50 ± 0.61 ^a^	30.00 ± 4.90 ^a^	28.00 ± 4.90 ^a^	5.00 ± 0.98 ^a^	24.00 ± 3.06 ^c^	0.0190 ± 0.0018 ^b^
GOG	11.91 ± 0.75 ^c^	113.50 ± 9.36 ^c^	92.78 ± 4.32 ^c^	24.15 ± 1.97 ^c^	6.87 ± 0.27 ^a^	0.0115 ± 0.0006 ^a^

Data are presented as the mean ± standard deviation (SD; *n* = 3). Groups sharing the same letter within each column are not significantly different from one another (one-way ANOVA followed by Tukey’s HSD post hoc test, *p* > 0.05). CG, control group; MG, H₂O₂ model group; GHG, Ginkgolide B + H₂O₂ group; GHG + Com C, Ginkgolide B + H₂O₂ + Compound C group; MG + Com C, H₂O₂ + Compound C group; GOG, Ginkgolide B-only group. CAT, catalase; GPx, glutathione peroxidase; SOD, superoxide dismutase; GSH, reduced glutathione; MDA, malondialdehyde; PC, protein carbonyl.

Antioxidant enzymes and GSH. The activities of CAT, GPx, and SOD, as well as GSH content, were markedly lower in the H₂O₂ model group than in the control group (all *p* < 0.05). Pretreatment with GB followed by H₂O₂ exposure (GB + H₂O₂) significantly increased CAT, GPx, and SOD activities and GSH content compared with the H₂O₂ model group (all *p* < 0.05); however, these values remained below those of the control group (*p* < 0.05). When AMPK was inhibited by Compound C prior to GB and H₂O₂ treatment (GB + H₂O₂ + Compound C), the GB-induced elevations of all four antioxidant parameters were abolished: CAT, GPx, SOD, and GSH levels were significantly lower than those in the GB + H₂O₂ group (all *p* < 0.05) and did not differ from the H₂O₂ model group (*p* > 0.05). Antioxidant activities and GSH content in the H₂O₂ + Compound C group did not differ significantly from those in the H₂O₂ model group or the GB + H₂O₂ + Compound C group (*p* > 0.05). The group treated with GB alone displayed antioxidant enzyme activities and GSH content comparable to those of the control group (*p* > 0.05), and all were significantly higher than those in the H₂O₂-treated groups (*p* < 0.05).

Oxidative stress markers. MDA content in the H₂O₂ model group was significantly higher than that in the control group (*p* < 0.05). GB pretreatment (GB + H₂O₂) significantly reduced MDA levels compared with the H₂O₂ model group (*p* < 0.05), although MDA levels remained higher than in the control group (*p* < 0.05). Following AMPK inhibition, MDA in the GB + H₂O₂ + Compound C group rebounded significantly relative to the GB + H₂O₂ group (*p* < 0.05) and did not differ from the H₂O₂ model group (*p* > 0.05). The MDA concentration in the H₂O₂ + Compound C group was significantly higher than that in the control and GB-alone groups (*p* < 0.05) but did not differ significantly from the H₂O₂ model or GB + H₂O₂ + Compound C groups (*p* > 0.05). In contrast to MDA, one-way ANOVA revealed a significant overall difference in PC content among the six groups (*F* = 5.692, *p* = 0.006). *Post hoc* Tukey’s test showed that most pairwise comparisons were not statistically significant. Tukey’s *post hoc* analysis revealed that only the H₂O₂ + Compound C group exhibited significantly higher PC levels than the control and GB-alone groups (*p* < 0.05). No significant differences were observed between the H₂O₂ model group and the GB + H₂O₂ group (*p* > 0.05), nor between the GB + H₂O₂ group and the GB + H₂O₂ + Compound C group (*p* > 0.05). The GB-alone group did not differ significantly from the control group (*p* > 0.05). These findings indicate that although GB effectively attenuated H₂O₂-induced lipid peroxidation, its effect on protein carbonylation was limited under the present experimental conditions.

### 3.3 Relative mRNA expression levels of AMPKα and Nrf2 in RPEs

One-way ANOVA revealed significant differences in the relative mRNA expression of both AMPKα and Nrf2 across the six experimental groups (all *p* < 0.05).

The mRNA levels of AMPKα and Nrf2 in the H₂O₂ model group were significantly downregulated compared with the control group (*p* < 0.05). Both were significantly elevated in the GB + H₂O₂ group compared with the H₂O₂ model group (*p* < 0.05). However, Nrf2 mRNA expression in the GB + H₂O₂ group showed no significant difference compared with the control group (*p* = 0.065). Following AMPK inhibition, the mRNA expression of AMPKα and Nrf2 in the GB + H₂O₂ + Compound C group significantly decreased compared with the GB + H₂O₂ group (*p* < 0.05), falling to levels comparable to those in the H₂O₂ model group. The mRNA expression levels of both genes in the H₂O₂ + Compound C group did not differ significantly from those in the H₂O₂ model group or the GB + H₂O₂ + Compound C group (*p* > 0.05). In the GB-alone group, neither AMPKα nor Nrf2 mRNA expression differed significantly from that in the control group (*p* > 0.05); both indices were significantly higher than in the H₂O₂ model and inhibitor-treated groups (*p* < 0.05). Details are shown in Table 3.

**Table 3:** Relative mRNA and protein expression levels of AMPKα and Nrf2 in RPEs.

Groups	AMPKα mRNA	Nrf2 mRNA	AMPKα (protein)	Nrf2 (protein)
CG	1.00 ± 0.00 ^c^	1.00 ± 0.00 ^bc^	1.00 ± 0.00 ^c^	1.00 ± 0.00 ^c^
MG	0.35 ± 0.02 ^a^	0.46 ± 0.04 ^a^	0.65 ± 0.05 ^a^	0.62 ± 0.05 ^a^
GHG	0.73 ± 0.04 ^b^	0.87 ± 0.05 ^b^	0.84 ± 0.06 ^b^	0.81 ± 0.05 ^b^
GHG + Com C	0.36 ± 0.04 ^a^	0.48 ± 0.05 ^a^	0.64 ± 0.05 ^a^	0.60 ± 0.06 ^a^
MG + Com C	0.28 ± 0.02 ^a^	0.37 ± 0.04 ^a^	0.54 ± 0.05 ^a^	0.51 ± 0.05 ^a^
GOG	1.05 ± 0.07 ^c^	1.08 ± 0.09 ^c^	0.98 ± 0.05 ^c^	0.99 ± 0.04 ^c^
F	231.215	115.858	50.238	64.588
*P*	< 0.001	< 0.001	< 0.001	< 0.001

Data are presented as the mean ± SD (*n* = 3). Groups sharing the same letter within each column are not significantly different from one another (one-way ANOVA followed by Tukey’s HSD post hoc test, *p* > 0.05). CG, control group; MG, H₂O₂ model group; GHG, Ginkgolide B + H₂O₂ group; GHG + Com C, Ginkgolide B + H₂O₂ + Compound C group; MG + Com C, H₂O₂ + Compound C group; GOG, Ginkgolide B-only group.

### 3.4 Relative protein expression of AMPKα and Nrf2 in RPEs

Western blot analysis was performed to further examine the changes in AMPKα and Nrf2 protein expression following the addition of Compound C. Significant differences in the relative protein expression of both AMPKα and Nrf2 were observed among all groups (all *p* < 0.05).

**Figure 3:**
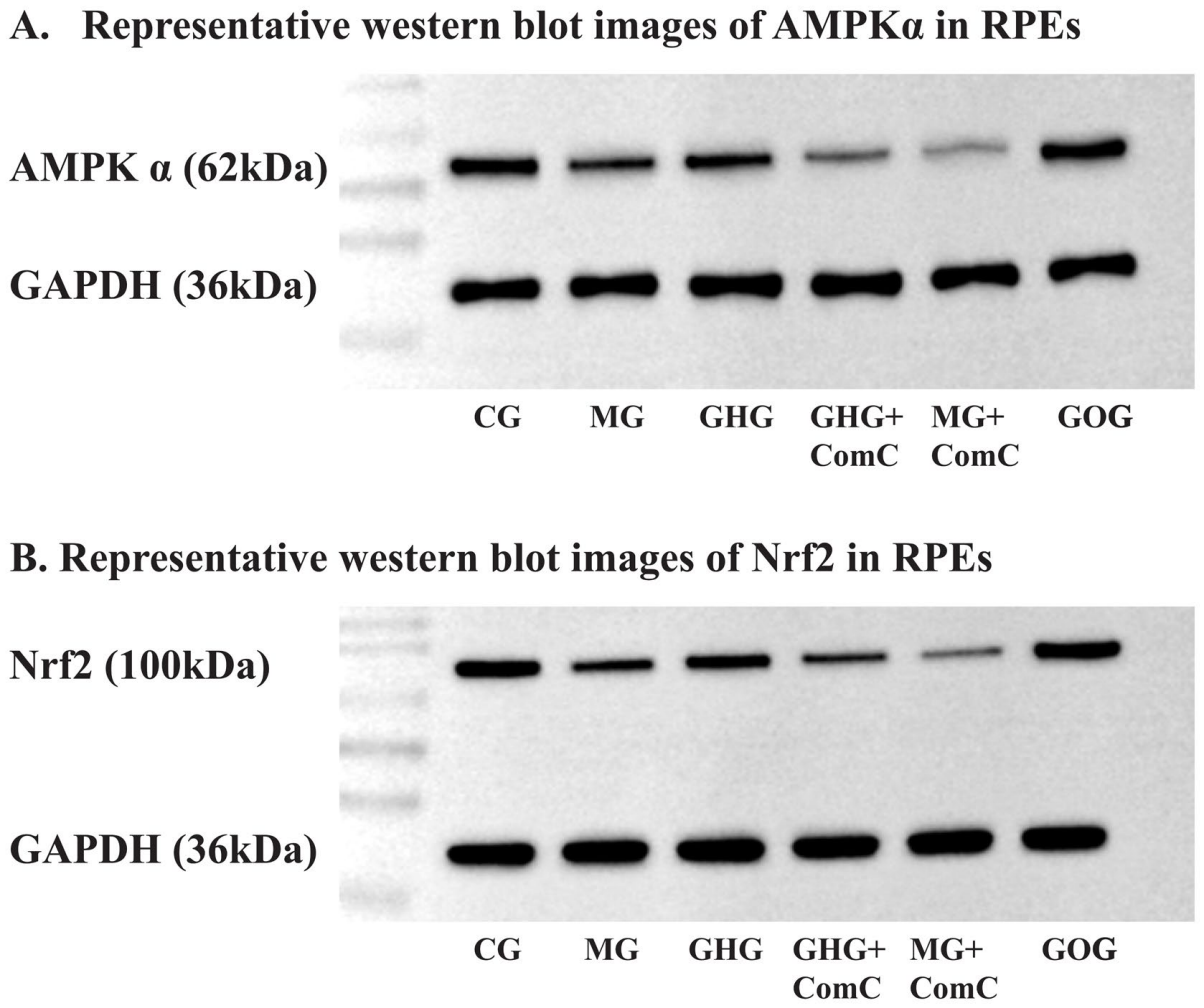
Representative Western blot bands of AMPKα and Nrf2 in RPEs. **(A)** Representative immunoblot of AMPKα (~62 kDa) and the loading control GAPDH (~36 kDa). **(B)** Representative immunoblot of Nrf2 (~100 kDa) and the loading control GAPDH (~36 kDa). AMPKα and Nrf2 were analyzed on two separate, parallel immunoblots (independent gels and PVDF membranes; one membrane for AMPKα and one membrane for Nrf2). For each membrane, the target protein and GAPDH were detected on the same membrane without stripping/reprobing using a two-step immunoblotting procedure (primary incubation followed by secondary incubation; no one-step primary–secondary co-incubation). Groups: CG, control group; MG, H₂O₂ model group; GHG, Ginkgolide B + H₂O₂ group; GHG + Com C, Ginkgolide B + H₂O₂ + Compound C group; MG + Com C, H₂O₂ + Compound C group; GOG, Ginkgolide B-only group.

The protein levels of AMPKα and Nrf2 in the H₂O₂ model group were significantly lower than those in the control group (*p* < 0.05). Both were significantly higher in the GB + H₂O₂ group than in the H₂O₂ model group, but remained lower than in the control group (*p* < 0.05). Following AMPK inhibition, the protein expression of AMPKα and Nrf2 in the GB + H₂O₂ + Compound C group significantly decreased relative to the GB + H₂O₂ group (*p* < 0.05), declining to levels comparable to those in the H₂O₂ model group. The protein expression levels in the H₂O₂ + Compound C group did not differ significantly from those in the H₂O₂ model group (*p* > 0.05). In the GB-alone group, the protein expression of AMPKα and Nrf2 showed no significant difference compared with the control group, and both were significantly higher than in the H₂O₂ model and inhibitor-treated groups (*p* < 0.05). Details are shown in Figure 3 and Table 3.

### 3.5 Cell viability of RPEs

As shown in Table 4, the CCK-8 assay was used to evaluate changes in cell viability after the addition of Compound C. Significant differences in cell viability were observed among all groups (*p* < 0.05). The cell viability in the H₂O₂ model group was significantly lower than that in the control group (*p* < 0.05). It was significantly higher in the GB + H₂O₂ group compared with the H₂O₂ model group, but remained lower than that in the control group (*p* < 0.05). Following AMPK inhibition, the cell viability in the GB + H₂O₂ + Compound C group decreased significantly relative to the GB + H₂O₂ group (*p* < 0.05), declining to a level comparable to that in the H₂O₂ model group. Furthermore, the cell viability in the H₂O₂ + Compound C group was significantly lower than that in the H₂O₂ model group (*p* < 0.05), indicating that AMPK inhibition alone further exacerbated H₂O₂-induced cell damage. No significant difference in cell viability was observed between the GB-alone group and the control group, and both were significantly higher than in the H₂O₂ model and inhibitor-treated groups (*p* < 0.05).

**Table 4:** Effects of Ginkgolide B on cell viability and apoptosis in H₂O₂-treated RPEs.

Groups	Cell viability (%)	Apoptotic cells (%)
CG	100.00 ± 0.00 ^d^	7.32 ± 0.19 ^a^
MG	53.82 ± 3.43 ^b^	37.25 ± 1.59 ^c^
GHG	78.30 ± 1.74 ^c^	22.21 ± 1.28 ^b^
GHG + Com C	56.00 ± 3.67 ^b^	35.86 ± 1.95 ^c^
MG + Com C	45.00 ± 4.29 ^a^	43.54 ± 2.69 ^d^
GOG	100.95 ± 2.41 ^d^	3.27 ± 0.55 ^a^
F	204.212	324.562
*P*	< 0.001	< 0.001

Data are presented as the mean ± SD (*n* = 3). Groups sharing the same letter within each column are not significantly different from one another (one-way ANOVA followed by Tukey’s HSD post hoc test, *p* > 0.05). CG, control group; MG, H₂O₂ model group; GHG, Ginkgolide B + H₂O₂ group; GHG + Com C, Ginkgolide B + H₂O₂ + Compound C group; MG + Com C, H₂O₂ + Compound C group; GOG, Ginkgolide B-only group.

### 3.6 Apoptosis of RPEs

**Figure 4:**
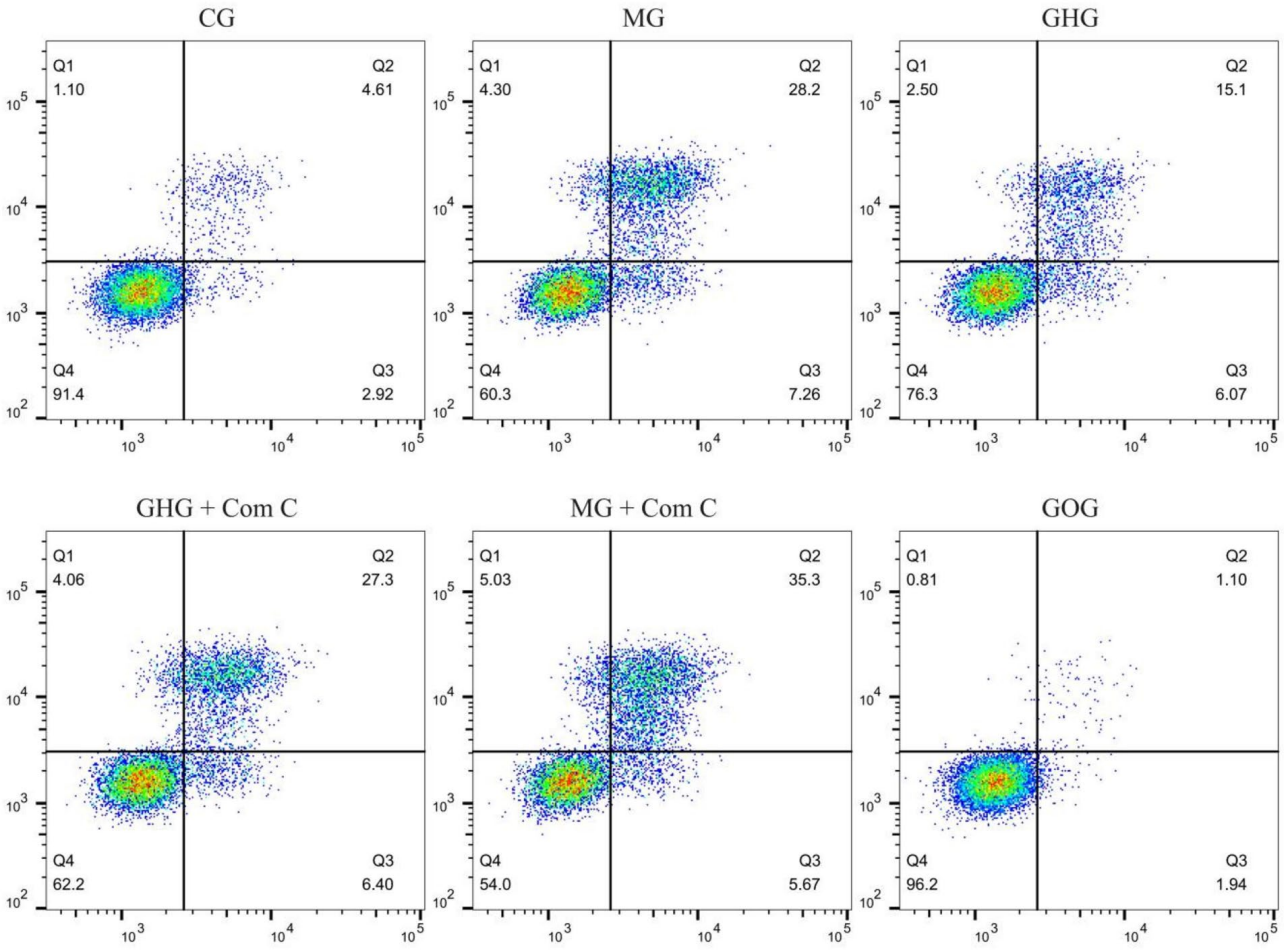
Percentage of apoptotic cells in RPEs (%).

As shown in Table 4 and Figure 4, flow cytometry was used to assess changes in the percentage of apoptotic cells after the addition of Compound C. One-way ANOVA revealed significant differences in the percentage of apoptotic cells among all groups (*F* = 324.562, *p* < 0.001).

The percentage of apoptotic cells in the H₂O₂ model group (37.25 ± 1.59%) was significantly higher than that in the control group (7.32 ± 0.19%, *p* < 0.001). Treatment with Ginkgolide B significantly reduced the percentage of apoptotic cells in the GB + H₂O₂ group (22.21 ± 1.28%) compared with the H₂O₂ model group (*p* < 0.001), though it remained significantly higher than that in the control group (*p* < 0.001).

Following AMPK inhibition, the percentage of apoptotic cells in the GB + H₂O₂ + Compound C group (35.86 ± 1.95%) increased significantly relative to the GB + H₂O₂ group (*p* < 0.001), rebounding to a level comparable to that in the H₂O₂ model group (*p* = 0.889). The percentage of apoptotic cells in the H₂O₂ + Compound C group (43.54 ± 2.69%) was significantly higher than that in the H₂O₂ model group (*p* = 0.005), indicating that AMPK inhibition alone further exacerbated H₂O₂-induced apoptosis. Similarly, the percentage of apoptotic cells in the GB-alone group (3.27 ± 0.55%) did not differ significantly from that in the control group (*p* = 0.080) and was actually lower, suggesting that GB alone may have a protective effect against baseline apoptosis.

## 4 Discussion

Our results demonstrate that GB effectively protects human RPEs from H_2_O_2_-induced oxidative stress and apoptosis, an effect that appears closely associated with modulation of the AMPK/Nrf2 signaling pathway. We observed that under intense oxidative stress, MG cells exhibited substantial mitochondrial damage, characterized by marked swelling, internal structural atrophy, and a tendency toward a spherical morphology. Conversely, in the GHG group subjected to GB intervention, the mitochondrial impairment was ameliorated, with a notable reduction in the number of damaged mitochondria. The treatment with GB significantly restored cell viability, attenuated oxidative damage by enhancing antioxidant enzyme activities and reducing lipid peroxidation, and consistently led to a marked decrease in the percentage of apoptotic cells. Crucially, this protective effect was associated with the upregulation of AMPKα and Nrf2 mRNA and protein expression. This study addresses the research gap highlighted in the introduction, suggesting that GB, unlike agents such as genipin or berberine, which primarily target a single pathway, may exert its protective effects by concurrently upregulating both AMPK and Nrf2, thereby offering a more comprehensive cytoprotective strategy against oxidative damage in RPEs.

The dual regulatory effect of GB on oxidative stress was clearly demonstrated in this experiment. In the Model Group (MG) treated with 200 μM H₂O₂ for 24 h, MDA levels were significantly elevated, indicating enhanced lipid peroxidation. However, PC levels did not differ significantly from those in the control group. This finding aligns with previous reports indicating that H₂O₂ increases MDA and decreases SOD activity in RPEs (11). The GHG significantly suppressed the rise in MDA, restoring it toward control levels. However, GB treatment did not significantly alter PC content under the present experimental conditions. Such antioxidant effects have also been reported in various models: GB reduced MDA and increased SOD activity in the hearts of diabetic rats, the hippocampi of hypoxic rats, and ischemic skin flaps (12–14). Further analysis of antioxidant defenses showed that key enzyme activities (CAT, GPx, SOD) were significantly decreased in the MG, suggesting dysfunction of the endogenous antioxidant system. GB intervention significantly restored these enzyme activities and increased GSH levels, indicating the reconstruction of defense mechanisms through enhanced expression of cellular antioxidant enzymes. Similar restoration of enzyme activities has been observed in studies of cerebral ischemia, vascular endothelial cells, and nerve cells, in which GB upregulated SOD, GPx, and CAT and increased GSH content, thereby inhibiting ROS production (9, 15, 16). Notably, Ginkgolide B also activated AMPK and upregulated Nrf2 expression, thereby driving the transcription of downstream antioxidant genes, such as HO-1 and NQO1, a mechanism experimentally validated in astrocytes and vascular endothelial cells (9, 14). In summary, GB was associated with improved redox homeostasis in RPEs through reductions in oxidative damage markers and enhancements of antioxidant defenses, thereby reducing oxidative damage products and bolstering endogenous antioxidant defenses, providing preliminary evidence for its potential application in oxidative stress-related retinal diseases.

In this experiment, H₂O₂-induced oxidative stress directly triggered apoptosis in RPEs, consistent with existing literature. Studies have shown that H₂O₂ can activate signaling pathways such as phosphatidylinositol 3-kinase (PI3K) / protein kinase B (Akt), c-Jun N-terminal kinase (JNK), and p38 mitogen-activated protein kinase (MAPK), leading to cell death; upregulation of miR-100 and phosphorylation of apoptosis-related proteins are involved, and inhibition of PI3K, JNK, or nuclear factor kappa-light-chain-enhancer of activated B cells (NF-κB) can significantly reduce oxidative cell death (17). Similarly, H₂O₂ treatment leads to decreased mitochondrial membrane potential and activation of cysteine-aspartic acid protease-3 (caspase-3), causing apoptosis, whereas the estrogen receptor beta (ERβ)- selective agonist GTx-822 can restore mitochondrial function and inhibit apoptosis, indicating that the mitochondrial pathway plays a dominant role in H₂O₂-induced RPEs apoptosis (18). Furthermore, peroxiredoxin 6 (PRDX6) overexpression significantly inhibited H₂O₂-induced cell apoptosis, suggesting that antioxidant proteins mediate cell survival via the PI3K/AKT pathway (19). Other studies directly confirmed that the natural flavonoid delphinidin significantly reduced H₂O₂-induced apoptosis by downregulating the expression of Bcl-2-associated X protein (Bax), cytochrome c, and caspase-3 (20). This evidence collectively constructs a causal chain linking oxidative stress, mitochondria, and apoptosis, providing a mechanistic basis for the high percentage of apoptotic cells observed in our experiment.

In this context, GHG significantly reduced the percentage of apoptotic cells (from 37.25 to 22.21%, *p* < 0.001), demonstrating its potent anti-apoptotic ability. Consistent with the literature, GB inhibited caspase-3 activation and significantly reduced cell apoptosis in retinal ganglion cells (21). This effect might be related to the preservation of mitochondrial integrity, possibly through modulation of mitochondrial permeability transition pore (mPTP) opening, although this requires further investigation, as well as maintaining mitochondrial membrane potential and blocking downstream apoptotic cascades. Notably, although the percentage of apoptotic cells in the GOG group (3.27%) appeared lower than that in the control group (7.32%), the difference was not statistically significant (*p* > 0.05), suggesting that GB is not only non-toxic but may also induce a “preconditioned” or “adaptively activated” state, equipping cells with higher intrinsic stress resistance even at baseline. Similar preconditioning effects have been reported for various natural antioxidants, including pre-treatment with delphinidin, which enhances anti-apoptotic gene expression prior to H₂O₂ exposure (20). Therefore, GB significantly reduced RPE apoptosis, potentially through multiple mechanisms involving antioxidant defense and mitochondrial protection.

In this study, GB significantly upregulated the transcription and protein levels of AMPKα and Nrf2, suggesting that its cytoprotective actions are potentially mediated by the AMPK-Nrf2 axis. The existing literature indicates that GB can activate the Akt-Nrf2-HO-1 signaling pathway, thereby enhancing antioxidant enzyme expression (22). More importantly, GB increased phosphorylated AMP-activated protein kinase (p-AMPK) levels and inhibited cell apoptosis in an ischemic stroke model, confirming its functional effect on AMPK activation (23). In astrocyte experiments, the protective effect of GB was completely abolished by the AMPK inhibitor Compound C, further proving the necessity of the GB-AMPK-Nrf2 triple pathway in defending against oxidative damage (9). Meanwhile, the exogenous oxidant bisphenol A (BPA) downregulated AMPK phosphorylation and inhibited Nrf2 expression, leading to an imbalance in RPEs antioxidant defense, a phenomenon consistent with the H₂O₂-induced decrease in AMPK/Nrf2 observed in our experiment (24). In RPEs models, C1q/tumor necrosis factor-related protein 9 (CTRP9) significantly alleviated high-glucose-induced oxidative stress and apoptosis by activating the AMPK/Nrf2 pathway, and inhibition of either AMPK or Nrf2 reversed its protective effect (25). Similarly, the natural diterpene Diosgenin also inhibited high-glucose-induced RPEs damage via the AMPK/Nrf2/HO-1 axis (26). These studies collectively indicate that AMPK is an upstream energy-sensing kinase that may promote Nrf2 nuclear translocation and drive the transcription of downstream antioxidant genes (e.g., HO-1, NQO1, SOD, CAT) through direct phosphorylation or indirect regulation of the Keap1-Nrf2 interaction (3, 27).

In our experiment, GB pretreatment significantly restored the activities of CAT, GPx, and SOD and increased GSH levels, indicating a direct manifestation of Nrf2-driven recovery of antioxidant gene expression. Furthermore, GB-induced AMPK upregulation may also promote cellular energy metabolism and mitochondrial function, inhibiting ROS generation and thereby blocking the signal transduction from oxidative stress to apoptosis, a mechanism that echoes the mechanism by which GB inhibits cell death via the AMPK-PTEN-induced putative kinase 1 (PINK1) pathway in ischemic brain injury (23). In summary, GB treatment was associated with dual inhibition of H₂O₂-induced oxidative damage and cell apoptosis, providing preliminary evidence for its potential application in retinal degenerative diseases.

*Ginkgo biloba* extract (GBE) activates the Nrf2-extracellular signal-regulated kinase (ERK) pathway in RPEs. However, this study systematically elucidates, at the level of a single natural component, GB, the complete signaling network encompassing upstream AMPK activation and downstream Nrf2-mediated antioxidant gene regulation, surpassing traditional strategies that target only Nrf2. Compared with confirmed AMPK activators such as metformin, this study further reveals that natural terpene lactones couple AMPK with Nrf2, providing new insights for developing more targeted natural drugs with fewer side effects.

### 4.1 Strengths and limitations

The present study has several strengths. First, it comprehensively evaluated the protective effects of Ginkgolide B against H₂O₂-induced retinal pigment epithelial cell injury using multiple complementary approaches, including biochemical assays, apoptosis analysis, transmission electron microscopy, qPCR, and Western blotting. Second, the inclusion of both Compound C-treated groups and a GB-alone group enabled a more systematic assessment of the potential involvement of AMPK signaling and the effects of GB under basal conditions. Third, the consistency among ultrastructural, molecular, and functional findings strengthens the overall reliability of the observed protective effects.

Nevertheless, several limitations should be acknowledged. Although Compound C was used to investigate the potential involvement of AMPK signaling, mechanistic conclusions were based primarily on pharmacological inhibition and should therefore be interpreted with caution, as chemical inhibitors may exhibit off-target effects. In addition, the specific role of Nrf2 was not directly examined using selective Nrf2 inhibition or genetic approaches such as gene silencing. While total Nrf2 mRNA and protein expression were measured, Nrf2 nuclear translocation, a key step in Nrf2 activation, was not directly assessed. Future studies employing nuclear–cytoplasmic fractionation, immunofluorescence staining, selective Nrf2 inhibitors, or genetic manipulation strategies will be necessary to further clarify the underlying mechanism. Finally, all experiments were conducted *in vitro* using ARPE-19 cells, and the protective effects of GB should be validated in animal models, including AMD models and genetically modified models targeting the AMPK/Nrf2 pathway.

## 5 Summary

In summary, GB treatment was associated with increased AMPKα and Nrf2 expression and improved antioxidant defense. Although pharmacological inhibition of AMPK attenuated these effects, further studies are needed to establish the precise mechanistic relationship between GB and AMPK/Nrf2 signaling. This protective effect was attenuated by the AMPK inhibitor Compound C, suggesting that AMPK/Nrf2 signaling may be involved in GB-mediated cytoprotection. However, because the present conclusions are based on pharmacological inhibition, additional studies employing genetic approaches and direct Nrf2-targeted interventions are required to confirm the underlying mechanism. Further studies incorporating *in vivo* models are warranted to substantiate these observations. This finding deepens understanding of the ocular protective mechanisms of GB and provides an important theoretical basis for its potential use as a promising natural agent to prevent and treat retinal degenerative diseases driven by oxidative stress, such as AMD.

## Data Availability

The raw data supporting the conclusions of this article will be made available by the authors, without undue reservation.

## Footnotes

1 Product page: https://www.chemicalbook.com/ProductChemicalPropertiesCB45856017.htm, catalogue number: CB45856017.
